# Supplementary material for: Estimating the potential biodiversity impact of redeveloping small urban spaces: the Natural History Museum’s grounds
Source: PeerJ. 2017 Oct 30;5:e3914. doi: 10.7717/peerj.3914 (PMC5667537; doi:10.7717/peerj.3914)
Supplement: Supplemental Information 1 [file peerj-05-3914-s001.docx]

**Supplemental Material for:**

**Estimating the potential biodiversity impact of redeveloping small urban spaces: the Natural History Museum's grounds**

Helen R. P. Phillips^1,2,3^, Sandra Knapp^2^, Andy Purvis^1,2^

^1^ Imperial College London, Silwood Park Campus, Buckhurst Rd, Ascot, SL5 7QN, U.K.

^2^ Natural History Museum, Department of Life Sciences, Cromwell Rd, London SW7 5BD, U.K

^3^ Current address: German Centre for Integrative Biodiversity Research (iDiv) Halle-Jena-Leipzig, Deutscher Platz 5e, 04103 Leipzig, Germany

**Corresponding Author:**

Helen R. P. Phillips^1,2,3^

Email address: [helen.phillips@idiv.de](mailto:helen.phillips@idiv.de)

***Supplemental Text S1***

A Supplementary Planning Document from the Royal Borough of Kensington and Chelsea (Royal Borough of Kensington and Chelsea, 2012) required the Museum to develop a unified scheme across the grounds to balance the competing demands from historical significance of the iconic Waterhouse building, the setting of the listed building, visitor amenity and use of space for events. To this end, an international design competition was held in Autumn 2013. The competition attracted 43 entries from around the world, reduced to six finalists in early 2014 and a single winner unanimously chosen by a jury, chaired by Ian Henderson CBE, in April 2014. The competition was won by the team Niall McLaughlin Architects and Kim Wilkie, whose plan contained an overarching continuous theme similar to that of the museum building, moving from “extinct” habitats in the east to current British habitats in the west (Figure 1b, also see <http://www.kimwilkie.com/london/natural-history-museum>).

It has been argued by critics of the proposal that the grounds harbour unusually high levels of biodiversity. Over 2800 species have been recorded in the WLG in the 21 years since its creation, in occasional structured surveys and more haphazard observations. Despite species having been recorded since 1995 when the WLG was created, new species continue to be added to the cumulative list of taxa recorded. Perhaps the most notable was that of the earthworm *Dendrobaena pygmaea*, not recorded in the UK for 32 years prior to its discovery in the WLG. Some previously unrecorded species have probably been able to colonise because of disturbance within and at the edge of the WLG (Ware et al.). For example, common cudweed (*Filago vulgaris*) and flixweed (*Descurainia sophia*), both vascular plants rare in London, were able to grow following disturbance from the building of the Darwin Centre in 2010 (Ware et al.). Species accumulation curves may be beginning to plateau in some taxa. For example, between 1995 and 2003, 100 species of algae were recorded, but only ten further species have been added since 2003. However, other taxa show no sign of saturation: 18 species of Araneae were recorded by 2003, for instance, but this had risen to 80 by 2015 (Ware et al.).

**References**

Royal Borough of Kensington and Chelsea. 2012. *Local Development Framework: Natural History Museum Grounds Design Guidance*. The Royal Borough of Kensington and Chelsea.

Ware C., Lowe M., Sivell D., Baker A., Bantock T., Barclay M., Carr G., Cooper L., Ellis L., Hall M., Hollowday E., Honey M., Dave J., Martin J., Notton D., Osborne D., Rundle A., Sherlock E., Tabor B., Thomas TJ., Thüs H., Tovey J., Wolseley P. *Further developments of the flora and fauna of the Wildlife Garden at the Natural History Museum, London -- Twenty years of species recording*.

***Supplemental Text S2***

Search terms for first query – 12^th^ October 2015

(``Species richness'' OR occurrence OR biodiversity) AND (Heath OR heathland OR Fen OR carr OR ``Secondary forest'' OR ``Secondary woodland'' OR woodland OR hedgerow OR ``urban park'' OR park OR ``broadleaved woodland'' OR grassland OR ``acid grassland'' OR ``calcareous grassland'' OR ``chalk grassland'' OR ``semi-improved grassland'' OR ``neutral grassland'' OR ``marginal vegetation'' OR pond OR wetland OR bog OR ``green roof'' OR mulch OR ``ephemeral vegetation'' OR ``short perennial vegetation'' OR orchard OR ferns OR cycad OR garden OR ``green space'') AND (U.K. OR United Kingdom OR Britain OR England OR Wales OR Scotland) AND (Urban OR Town OR City OR Village) AND (Plants OR Vegetation OR Invertebrates OR Arthropods OR Arthropoda OR Insects OR ``above-ground biodiversity'' OR ``below-ground biodiversity'')

***Supplemental Text S3***

Search terms for second query – 28^th^ October 2015

(``Species richness'' OR occurrence OR biodiversity OR abundance OR ``species density'' OR diversity OR richness) AND (U.K. OR United Kingdom OR Britain OR England OR Wales OR Scotland) AND (Urban OR Town OR City OR Village)

***Supplemental Text S4***

Theory predicts that species density should scale with fragment area at a slope of 0.1, however, previous analysis has shown that the slope was 0.07 (95% confidence interval: 0.048 - 0.11), thus the analysis was repeated with z = 0.07. Species density (per 10m^2^) significantly varied between habitat types (χ^2^ = 377.23, d.f. = 12, p < 0.01). Assumption 1 yields an increase of 12.17%. Under Assumption 2, the increase is estimated to be 13.5%.

***Supplemental Figure S5***

The number of times each percentage change was obtained in the sensitivity analysis, under (a) Assumption 2 and (b) Assumption 3 (see main text for detailed information on Assumptions).


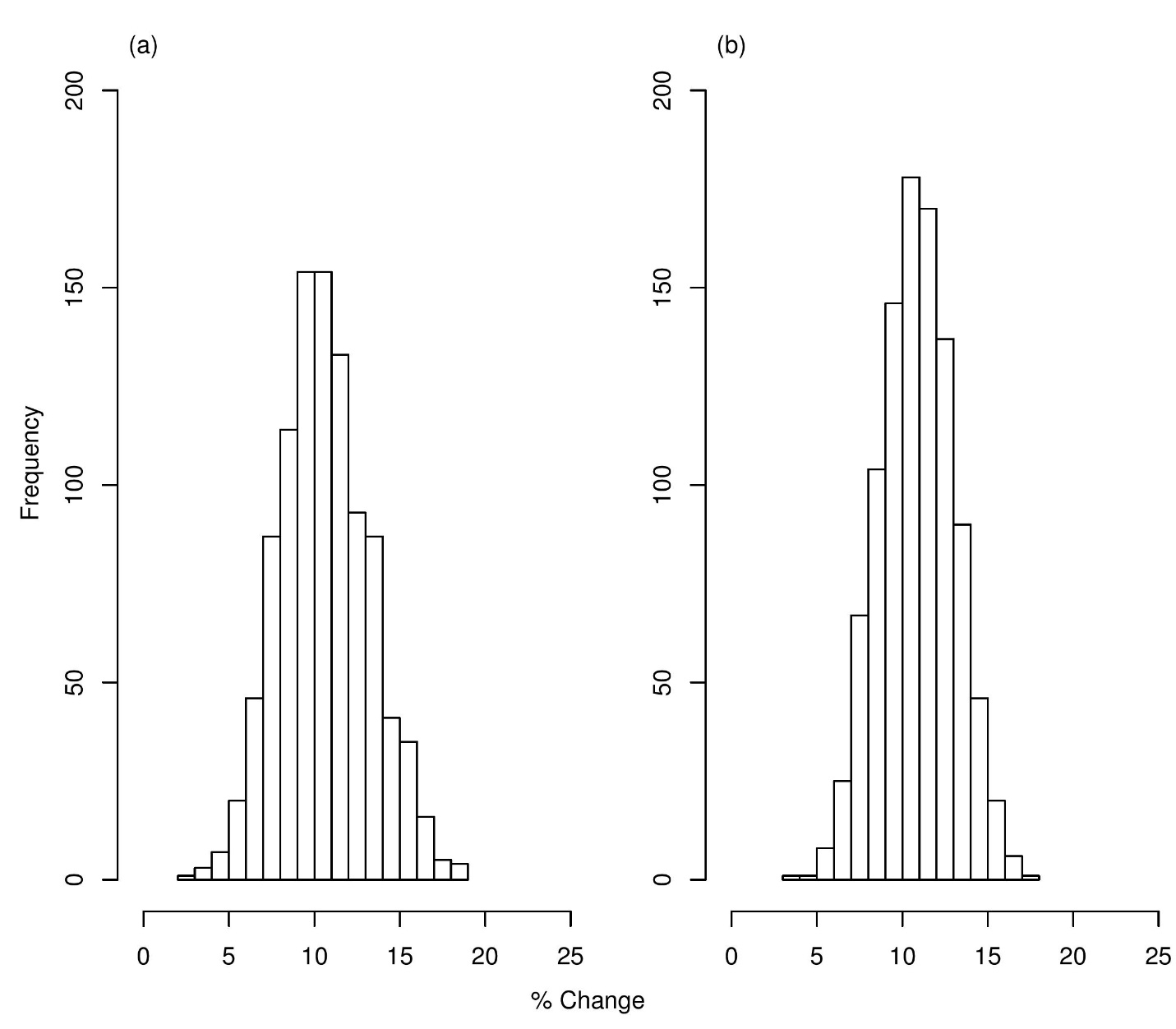


A random sample was taken from the distribution of each habitat coefficient obtained from models based on the different assumptions, and the overall biodiversity gain or loss was calculated. This was repeated 1000 times. None of the runs resulted in a loss of species richness under either Assumption.
